# Pharmaceutical Potential Evaluation of Damask Rose By-Products from Volatile Oil Extraction

**DOI:** 10.3390/plants13121605

**Published:** 2024-06-09

**Authors:** Nutthawut Charoimek, Piyachat Sunanta, Tibet Tangpao, Ratchuporn Suksathan, Wisinee Chanmahasathien, Sasithorn Sirilun, Kuo-Feng Hua, Hsiao-Hang Chung, Sarana Rose Sommano, Taepin Junmahasathien

**Affiliations:** 1 Department of Pharmaceutical Science, Faculty of Pharmacy, Chiang Mai University, Chiang Mai 50200, Thailand; nutthawut_charoimek@cmu.ac.th (N.C.); wisinee.c@cmu.ac.th (W.C.); sasithorn.s@cmu.ac.th (S.S.); 2 Plant Bioactive Compound Laboratory (BAC), Faculty of Agriculture, Chiang Mai University, Chiang Mai 50200, Thailand; piyachat.sun@cmu.ac.th (P.S.); tibet.t@cmu.ac.th (T.T.); 3 Research Unit for Innovation in Responsible Food Production for Consumption of the Future (RIFF), Multidisciplinary Research Institute, Chiang Mai University, Chiang Mai 50200, Thailand; 4 Office of Research Administration, Chiang Mai University, Chiang Mai 50200, Thailand; 5 Queen Sirikit Botanic Garden, The Botanical Garden Organisation, Chiang Mai 50180, Thailand; r_spanuchat@yahoo.com; 6 Department of Biotechnology and Animal Science, National Ilan University, Yilan City 260, Taiwan; kuofenghua@gmail.com; 7 Department of Horticulture, National Ilan University, Yilan City 260, Taiwan; hhchung@niu.edu.tw; 8 Center of Excellence in Agro Bio-Circular-Green Industry (Agro BCG), Faculty of Agro-Industry, Chiang Mai University, Chiang Mai 50100, Thailand; 9 Department of Plant and Soil Sciences, Faculty of Agriculture, Chiang Mai University, Chiang Mai 50200, Thailand

**Keywords:** antioxidant, antimicrobial, hydro-distillation, *Rosa damascena* Mill., rose volatile processing

## Abstract

Despite its well-known fragrance in cosmetics and medicine, a complete understanding of the phytochemical properties within by-products generated during commercial extraction of Damask rose remains elusive. Cultivated in Thailand for their essential oil, Damask rose varieties, including Mon Dang Prasert, Mon Klai Kangwon, and Bishop’s Castle, share phenylethyl alcohol (57.62–61.11%) as the dominant component, which is responsible for their characteristic floral, sweet, rosy, and bready aroma. Through a circular hydro-distillation process, three different by-product fractions, including distilled water (D), hydrosol (H), and rose dreg (R), were recovered. Subsequently, we assessed their pharmaceutical potential, including the antioxidant, antimicrobial, anti-inflammatory, and anti-melanogenesis properties of these residual substances. The H fraction displayed the highest total phenolics (10.56 mgGAE/g) and flavonoids (6.93 mgCE/g) and significant antioxidant activity (IC_50_, 0.67–0.97 µg/mL). While the H fraction inhibited melanin formation at 50 μg/mL, the R fraction of MK (100 μg/mL) surprisingly promoted melanin production in B16-F10 cells. Nevertheless, the antimicrobial assay against *Staphylococcus aureus*, *Cutibacterium acnes*, *Staphylococcus epidermidis*, *Pseudomonas aeruginosa*, *Escherichia coli*, and *Candida albicans* revealed no antimicrobial activity in any fraction. Murine macrophage stimulation (J774A.1) with lipopolysaccharide revealed no anti-inflammatory effects from the by-products, as measured by IL-1β production. In summary, the H fraction exhibited the highest level of phenolic and flavonoid contents, as well as antioxidant and anti-melanogenesis activities. Therefore, this by-product is a desirable choice for the development of value-added products such as functional food, cosmetics, and pharmaceutical products.

## 1. Introduction

Damask rose, scientifically known as *Rosa damascena* Mill., is native to Europe and Middle Eastern countries and is cultivated mainly in Iran and Turkiye [1,2]. In addition, this ornamental plant is widely favoured for cultivation in residential landscapes, gardens, and interior spaces [3,4]. For instance, commercial varieties such as Svezhen and Iskra are extensively bred and valued for their significance in the production of essential oil [5,6,7]. Beyond its versatile use in products, including rose essential oil, rose water, rose concrete, and rose absolute, rose essential oil plays a pivotal role as a fundamental ingredient in the formulation of flavourings, cosmetics, and various health-related products [8]. Globally, the annual production of rose essential oil is estimated to be around 4.5 tons, and forecasts suggest a 6.8% average yearly growth rate, reaching a market value of USD 442 million from 2019 to 2025 [9,10,11]. The primary components of rose essential oil consist of alkanes, alcohols, phenols, terpenes, and terpenoids. This oil contains some significant alkanes, namely nonadecane, eicosane, heneicosane, heptadecane, and octadecane. Additionally, the major terpene and terpenoid components presented are phenylethyl alcohol, citronellol, geraniol, nerol, linalool, and farnesol [12,13]. It also demonstrates pharmacological advantages, including antioxidant, antibacterial, antimicrobial [14,15], anti-inflammatory [16], anti-cancer [17], and anti-HIV properties [18].

The conventional hydro-distillation method is widely recognised as the most prevalent and economically efficient technique for extracting essential oil [19]. Following this extraction procedure, significant quantities of the aqueous phase (known as distillate), plant dregs, and non-vapourised aqueous phase (also referred to as hydrosols) were disposed of at the end of extraction [20]. Currently, there has been a notable increase in the demand for botanicals as ingredients in pharmaceutical products, including skin formulation and dietary supplements. However, the extraction of functional ingredients from agricultural by-products has attracted considerable attention in light of the Bio-Circular-Green (BCG) Economic Model. By-products obtained from the distillation of roses serve as valuable sources of natural products [21]. These by-products have demonstrated various medicinal benefits, including alleviating digestive disorders, promoting kidney and liver function, and preventing skin and eye irritation [22]. Moreover, the utilisation of rose by-products extends to diverse applications, encompassing composting, animal feed, natural dye production, biogas generation, pollutants biosorption, and the extraction of phenolics and pectic polysaccharides [23].

In Thailand, the cultivation of Damask rose for the fragrance industry is in the initial phase. The three mainly cultivated varieties are Mon Dang Prasert (MD), Mon Klai Kangwon (MK), and Bishop’s Castle (BC) [24]. The rising market value of rose essential oil, coupled with the low extraction yield, resulted in a notable increase in the demand for rose cultivation. Approximately 12,000 tons of rose petals are commercially extracted, resulting in the generation of a massive amount of by-products that are typically disposed of in landfills, leading to many serious environmental issues [25,26]. However, there is a lack of research on the aromatic profiles, phytochemical properties, and pharmacological effects of these by-products. In alignment with the principles of the bio-circular zero-waste approach, this research aims to extract and analyse the phytochemical properties and evaluate pharmaceutical potential relevant to skincare from this Damask rose variety. The overall outcome of this study is expected to contribute not only to the ingredients of novel products through the utilisation of by-products but also to represent a significant advancement toward achieving sustainable development objectives.

## 2. Results and Discussion

### 2.1. Flower Morphological Characteristics

Throughout the initial taxonomical examination, it was found that roses from three different varieties had consistent morphological characteristics. These roses were identified as Damask roses (*R. damascena* Mill.), a result of deliberate crossbreeding [27,28]. Notably, the flowers exhibited a wide spectrum of colours, ranging from delicate shades of pink to deep red, accompanied by a robust and captivating fragrance upon blooming. From our glance, the MK cultivar stood out for possessing the most potent fragrance, while the BC rose flowers exhibited a unique olfactory signature. Further insights into the morphological attributes, moisture content percentage, and fragrance compositions of these roses are elucidated in Table 1, providing a comprehensive understanding of their distinctive morphological and aroma characteristics. The botanical origins of the Damask roses were elucidated by Iwata et al. [29], revealing a complex lineage involving the maternal ancestor *R. moschata* and two successive crossings (*R. moschata* × *R. gallica*) × *R. fedschenkoana* Regel. Generally, the morphological characteristics of these roses are flowering shrubs, characterised by an upright stem adorned with sharp, curved thorns, which attains a substantial height of 1–2 m. Notably, its flowering potential is remarkable, with the capability to yield between 500 and 600 flowers annually, contingent upon reaching a minimum age of four years [30,31]. The flowers themselves exhibit a captivating spectrum of colours, ranging from delicate light pink to medium pink and culminating in rich, dark red hues. The cultivation of Damask roses in Thailand encompasses three prominent varieties, including MD, MK, and BC. The MD is distinguished by its vibrant red petals; this particular rose has established a longstanding presence in Thailand, qualifying it as an ancient and culturally significant floral specimen originally from the Middle East [32]. The MK, distinguished by its delicate pink blossoms and strong fragrance, is an ancient and heirloom variety that has been present in Thailand and is found across various global regions, including India, the Central East, and Europe, particularly in Iran, Turkiye, and Bulgaria [33]. Additionally, the BC, originating from England, emerges as a noteworthy variety suitable for cultivation in the Thai climate, exhibiting resilience against diseases and pests [34]. These diverse Damask rose varieties contribute to the rich horticultural landscape in Thailand, reflecting both historical significance and adaptability to local conditions.

All in all, this study demonstrates distinct physical and sensory characteristics among the Damask rose varieties investigated. To further elucidate these findings, it is imperative to complement the descriptive sensory analysis with a more in-depth examination of the aromatic compound profiles. Additionally, this research aims to analyse the aromatic profile of these roses at various stages of development that may provide valuable information for both farmers and industry stakeholders, ultimately aiding in the selection of optimal rose varieties for commercial applications.

### 2.2. Aromatic Compound Profiling of Fresh Damask Rose and Volatile Fractions

**Figure 1:**
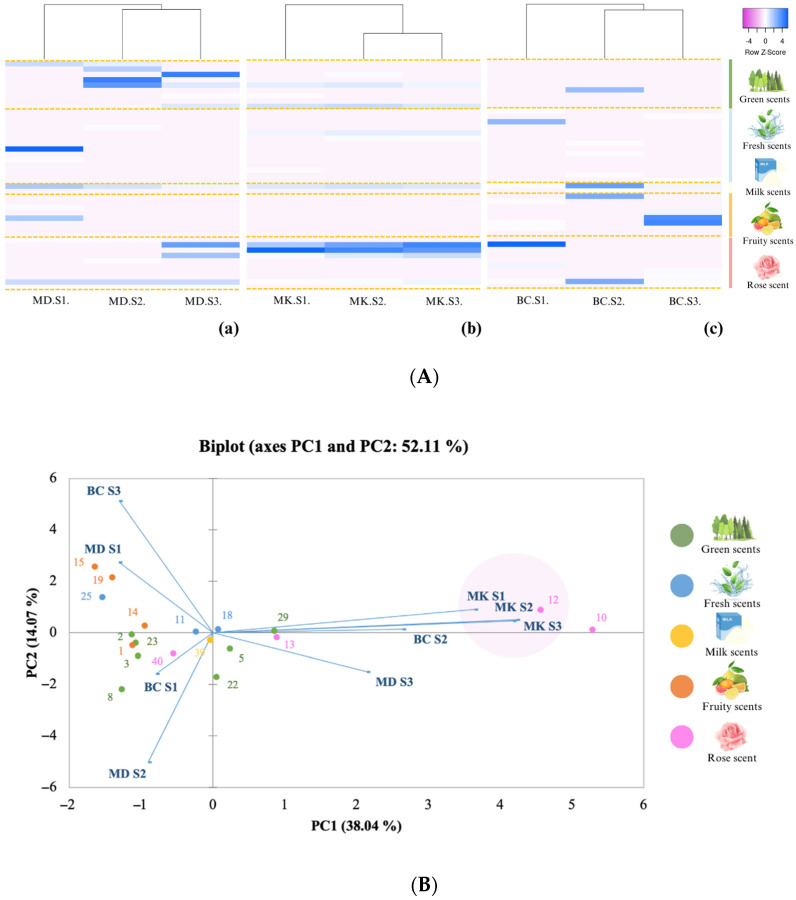
Heat map plot clusters of Damask rose varieties, (**a**) Mon Dang Prasert (MD), (**b**) Mon Klai Kangwon (MK), and (**c**) Bishop’s Castle (BC), based on their odour descriptor of aromatic compounds in the bud stage (S1), starting to bloom stage (S2), and full bloom stage (S3) (**A**). The principal component analysis (PCA) between three Damask rose varieties, including Mon Dang Prasert (MD), Mon Klai Kangwon (MK), and Bishop’s Castle (BC), and their aromatic compounds based on their odour descriptor of aromatic compounds in the bud stage (S1), starting to bloom stage (S2), and full bloom stage (S3) (**B**).

The chemical profiling of each Damask rose variety discerns distinct patterns (Supplementary Table S1). The volatile profiling showed that the major compounds found in the MK were phenylethyl alcohol and citronellol, which were closely aligned across all developmental stages. In contrast, MD at stages 1 and 2 and BC at stages 1 and 3 formed a separate grouping based on the presence of 9-nonadecene and eicosane as prominent chemical constituents. The aromatic profiling data were also elucidated across multiple developmental stages, as illustrated in Figure 1A. Utilising heat map analysis, the aromatic profiles discern the dynamic nature of rose fragrances during different stages of development for each variety. In the MD, stage 1 was characterised by fresh scents, while stages 2 and 3 exhibited predominant green and rose scents. Conversely, the MK rose variety demonstrated a consistent classification of scents across all stages, encompassing rose, milk, and green fragrances. In the case of the BC rose variety, stage 1 exhibited distinct rose and fresh scents, stage 2 presented rose and milk scents, and stage 3 featured a pronounced presence of fruity scents. The relationship of the aromatic compounds in the three Damask rose varieties, including MD, MB, and BC, is shown in Figure 1B. The variables accounted for a total of 52.11%, deposited for 38.04% in PC1 and 14.07% in PC2. The PCA indicated that the MK samples at each stage could not be distinguished based on volatile chemicals. The MK samples were grouped together and isolated from other samples by phenylethyl alcohol and citronellol, which represented the scent of rose. The volatile components of roses were highly concentrated during the full blooming period for the MD. Initially, the BC emitted a rose fragrance in the first and second stages, but with varying volatile components. However, at the fully bloomed stage, the scent transformed into a fruity and fresh aroma. Our findings suggested that the aromatic profile of MK found in this study closely resembled the findings of Karami et al. [35], which identified phenyl ethyl alcohol, β-citronellol, α-pinene, benzyl alcohol, and geranyl acetate as the predominant compounds in Iranian Damask roses. Moreover, the investigation of volatile compounds in Chinese Damask rose identified 89 chemicals detected throughout all stages of blooming [36], whereas the full-blooming stage exhibits the highest diversity and relative abundance of chemicals such as β-myrcene, pentadecane, ethylbenzene, and o-xylene. The study of Dobreva and Nedeltcheva-Antonova [37] also reported that the essential oil of Damask rose consisted of monoterpene alcohols, such as citronellol, geraniol, and nerol, along with a stearopten fraction featuring nonadecane, nonadecene, heneicosane, and heptadecane. These findings offered a comprehensive understanding of the aromatic complexity within distinct rose varieties.

**Figure 2:**
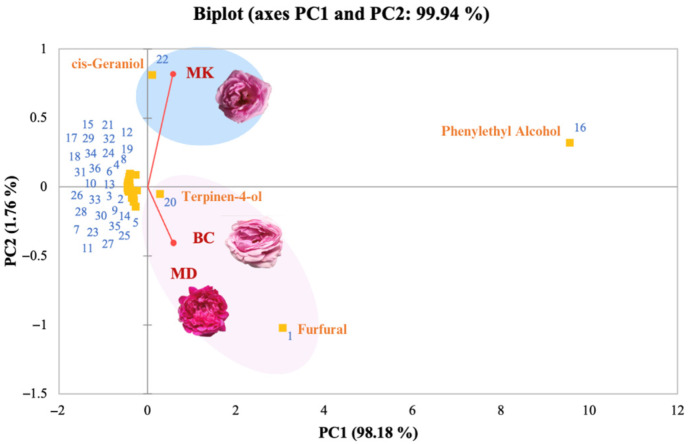
The principal component analysis (PCA) in volatile fractions of three Damask rose varieties, Mon Dang Prasert (MD), Mon Klai Kangwon (MK), and Bishop’s Castle (BC), based on their chemical composition of aromatic compounds (presented in Supplementary Table S2).

From this work, our analysis of Damask rose varieties reveals distinct fragrance profiles characterised by specific chemical components across different developmental stages. Phenylethyl alcohol and citronellol emerge as prominent contributors to the rose fragrance of the MK variety, maintaining consistent scent classifications throughout development. Conversely, MD and BC varieties exhibit varying fragrance profiles, transitioning from fresh and green scents to rose and fruity aromas, respectively. We selected stage 3 from three Damask rose varieties for the extraction. This was chosen due to its ability to emit a clear rose scent and its greater stability compared to the other stages. Damask rose is crucial for the essential oil extraction industry due to its unique odour. Flowers at the selected stage were then used for the extraction of the volatile fraction as the product with commercial need. The chemical analysis of the volatile compounds presented in three rose varieties revealed a rich and diverse composition. A total of 36 aromatic compounds were identified, as detailed in Supplementary Table S2. Employing statistical methods for categorisation, the primary components were investigated across various variables, including the chemical makeup of aroma compounds and the specific rose varieties under examination (Figure 2). Note that the analysis highlighted the prominence of phenylethyl alcohol in the three rose varieties, with concentrations found to be significantly high. These phenylethyl alcohols can represent floral, sweet, rose, and bready notes [38]. Upon further classification, the varieties were distinctly grouped into two categories, where BC and MD varieties exhibited similar chemical compositions. The foundational chemical constituents guiding this classification included furfural, known for its fresh, sweet, and almond characteristics [39], as well as terpinen-4-ol, evoking similarities to turpentine oil and lavender oil [40]. In contrast, the MK variety was discerned based on cis-geraniol, characterised by a sweet, floral, and rosy aroma and notably higher quantities compared to other varieties [41].

Nevertheless, a substantial quantity of by-products was generated throughout the extraction process, which are typically disposed of in landfills. These by-products might cause various kinds of environmental issues. However, some scientific studies indicate that residual rose dregs from essential oil extraction contain numerous health benefits that are valuable for the food and feed industries.

### 2.3. Phytochemical Analysis of Damask Rose By-Products

Table 2 displays the bioactive compounds and antioxidant activity of the three by-product fractions from Damask rose. The hydrosol fraction exhibited a significantly higher total flavonoid content than the other fractions. The HMD contains the largest amount of flavonoids, specifically 6.93 mgCE/g. The hydrosol fraction contained the highest amount of total phenolics, and HMD and HMK showed the highest quantities at 10.08 and 10.56 mgGAE/g, respectively. The results of the ABTS antioxidant properties presented that the RMD sample exhibited the highest free radical inhibition percentage at 65.80%, the HMD sample revealed an IC_50_ value of 0.97 μg/mL, while the HMD sample gave the highest antioxidant activity at 0.12 mg TE/g. Similarly, the DPPH assay showed the highest inhibition at 60.85% in the RMK, while HMD displayed the highest IC_50_ value of 0.95 μg/mL, and HMD demonstrated the antioxidant potential with a value of 0.12 mgTE/g dried sample. The assessment of antioxidant activity through the FRAP method revealed that the highest inhibition percentage was highest in the RMK sample at 58.24%, and the HMD sample displayed a noteworthy IC_50_ value of 0.67 μg/mL. However, HMD showed the most antioxidant activity at a value of 3.89 mgTE/g. A comprehensive investigation was undertaken to examine and compare the outcomes of essential oil extraction from three distinct rose varieties, MD, MK, and BC, in the research of Prapalert and Phottraithip [42]. The result of free radical scavenging activity showed that the DPPH method revealed noteworthy findings. Specifically, the extracts exhibited IC_50_ values of 7.40, 25.00 and 30.00 μg/mL, respectively, with vitamin C and Trolox serving as standards. Furthermore, the total phenolic contents were 606.66, 101.93, and 92.31 mgGAE/g extract for the three respective rose varieties. Additionally, the study explored the total flavonoid content, reporting values of 178.76, 42.99, and 38.62 mg quercetin equivalent (QE)/g extract, respectively. Hence, it was evident that the ethanolic extracts derived from floral parts of all three rose varieties demonstrated superior inhibitory effects on both total phenolic content and total flavonoid content in comparison to the outcomes obtained with by-product fractions resulting from the extraction process. In the research conducted by Abdel-Hameed et al. [43], a thorough exploration was undertaken to investigate the biological and phytochemical properties of the by-product derived from rose water of Taif rose (*R. damascena trigintipetala* Dieck). This by-product was obtained as a residue subsequent to the hydro-distillation process. The free radical scavenging of the rose water by-product, as assessed through the DPPH radical method, was reposted with an IC_50_ value of 23.72 ± 0.36 µg/mL. Furthermore, the by-product demonstrated a remarkable total antioxidant capacity by phosphor-molybdenum method, with a quantified value at 329.53 ± 18.75 mg ascorbic acid equivalent/g extract, and exhibited significant reducing power activity at 211.31 ± 2.79 mg ascorbic acid equivalent/g dry extract. Furthermore, Dina et al. [44] performed a study with the aim of investigating the possible benefits of a concentrated polyphenolic extract obtained from the by-product of *R. damascena* hydro-distillation. The analysis revealed significant findings regarding the aqueous residue obtained from rose hydro-distillation. The residue showed high amounts of phenolic and flavonoid content, measured at 260 mgGAE/g extract and 80 mgQE/g extract, respectively. In addition, the liquid remaining after the process had a notable level of antioxidants, which was demonstrated by its ability to effectively neutralise DPPH and ABTS radicals. The concentration required to achieve a 50% reduction in these radicals was reported at 25.4 μg/mL and 8.7 μg/mL, respectively. In addition, the research by Trendafilova et al. [45] investigated the antioxidant potential of dry rose ethanol (DRE) extract and re-extracted with ethyl acetate (EAE) from *R. damascena* flowers. The study found that EAE exhibited significantly higher total phenolic and flavonoid contents compared to DRE, with values of 680.48 mgGAE/g extract and 482.26 mg rutin equivalents (RE)/g extract, respectively, as compared to 212.19 mgGAE/g extract and 135.28 mgRE/g extract for DRE. Additionally, analysis of antioxidant activities, including DPPH, ABTS, and FRAP, revealed that EAE displayed superior antioxidant properties when compared to DRE. This study revealed that by-products generated from the extraction of the Damask rose exhibit antioxidant potential. Essentially, the H fraction demonstrated particularly high levels of flavonoids and antioxidant activity. The next step was to identify the specific phenolic and flavonoid compounds responsible for this observed antioxidant effect.

### 2.4. Quantitative Analysis of Phenolic and Flavonoid

HPLC has been used to determine the phenolic and flavonoid compounds in the H and R of three different kinds of Damask rose (MD, MK, and BC). Table 3 displays different characteristics of the fractions and varieties. For gallic acid, a significant phenolic component, differences in concentration among the fractions were determined, with the HMK fraction having the highest level (14.17 mg/g). In contrast, epicatechin was not found in any of the fractions. The concentration of catechin/caffeic acid differed, and the MK variety had the highest level in both the H and R fractions. The HBC showed greater amounts of naringin, another flavonoid, compared to the other varieties (3.38 mg/g). The HMD fraction had the greatest quantity of p-coumaric acid (7.38 mg/g) among all the measured fractions. The quantity of rosmarinic acid was the highest (6.58 mg/g) throughout different fractions and varieties. Notably, the HBC exhibited the greatest level of vanillic acid (4.07 mg/g). Nevertheless, o-coumaric acid was not present in all fractions, while quercetin was only found in RMK and RBC. The resultant analysis corresponds with previous studies. Baydar and Baydar [21] used an HPLC system to examine phenolic components in methanolic extracts of *R. damascena* Mill flowers, including both fresh and processed flowers. The analysis indicated there was evidence of gallic acid content of more than 27.00 mg/g in processed flowers, quercetin up to 0.40 mg/g in processed flowers, and syringic acid at 0.54 mg/g in processed flowers. Interestingly, both the fresh and spent flower extracts lacked some phenolic acids, namely caffeic, chlorogenic, p-coumaric acid, and ferulic acids. Kumar et al. [46] discovered the presence of polyphenols, specifically gallic acid, quercitrin, quercetin, myricetin, rutin, and kaempferol, in both fresh flowers and dregs (the residue left after the industrial distillation of rose oil) of *R. damascena*. However, catechin, epicatechin, m-coumaric acid, and apigenin were not detected. Furthermore, the diversity and excellence of identified chemical composition in plant extracts are influenced by various factors, including internal factors such as genetic variation [47], as well as external factors such as growth conditions, processing methods, and extraction techniques [48]. For instance, the examination of Memariani et al. [49] discovered phenolic components in hydroalcoholic extracts of *R. damascena* obtained from two different places, namely Tabriz and Kashan. The analysis uncovered significant differences in the content of phenolic chemicals between both locations. The absence of caffeic acid, ferulic acid, and p-coumaric acid in both samples was highly significant, suggesting that the phenolic composition of the two rose sources differed significantly.

### 2.5. Antimicrobial Activity

This investigation focused on evaluating the antimicrobial efficacy of H and R fractions from the three rose varieties (MD, MK, and BC) against the proliferation of *S. epidermidis*, *S. aureus*, *C. acnes*, *E. coli*, *P. aeruginosa*, and *C. albicans*, as shown in Table 4. The determination of MIC and MBC or MFC was employed as the methodology. Intriguingly, the results indicated that both H and R fractions, functioning as by-products, exhibited an inability to impede the growth of the microorganisms. This observation was notably contrasted with the antibiotic, clindamycin hydrochloride (Clinda M; R.P.C. International Co., Ltd., Bangkok, Thailand), and penicillin sodium (Penicillin G; General Drugs House. Co., Ltd., Bangkok, Thailand), suggesting a disparity in antimicrobial activity between the experimental fractions and established antibiotics. Prapalert and Phottraithip [42] conducted a comprehensive investigation to assess the inhibitory effects of crude extracts obtained from roses of three distinct cultivars on the growth of *Proteus mirabilis*, *E. coli*, *S. aureus*, and *Bacillus cereus*, employing the agar well diffusion method. The study unveiled noteworthy outcomes, demonstrating the capacity of crude rose extracts to impede the growth of two Gram-positive bacteria, specifically *S. aureus* and *B. cereus*. Moreover, inhibitory effects were observed against the growth of *P. mirabilis*, a Gram-negative bacterium. Notably, the investigated crude extracts exhibited limited efficacy in inhibiting the growth of *E. coli*, a Gram-negative bacterium. A study by Trendafilova, Staleva, Petkova, Ivanova, Evstatieva, Nikolova, Rasheva, Atanasov, Topouzova-Hristova, and Veleva [45] also discovered that the dry rose extract from *R. damascena* flowers exhibited strong antimicrobial activity against several strains of bacteria, including *C. acnes*, *S. aureus*, and *S. epidermidis*, but showed no activity against *C. albicans*. The research by Bayhan et al. [50] revealed no reduction in colony counts of the *R. damascena* hydrosol in the hand gel formulation. The research conducted by Maruyama et al. [51] studied the inhibitory effects of diluted hydrosol prepared from *R. damascena* on neutrophil adhesion and its antimicrobial activity. The study showed significant findings, demonstrating that the rose hydrosol exhibited a notable inhibitory impact on the hyphal growth of *C. albicans* at a concentration of approximately 2.2%. Phenolics, such as quercetin and kaempferol, possess antibacterial characteristics and are significant components in the hydroethanolic extract of *R. damascena*, as stated by Chroho et al. [52]. Nevertheless, our investigation unveiled that the by-product extracts did not demonstrate efficacy in inhibiting harmful germs. The ineffectiveness of the treatment may be due to the insufficient concentration of extracts utilised in the experiments, which may not have been strong enough to have antibacterial effects.

### 2.6. Anti-Inflammatory Activity

**Figure 3:**
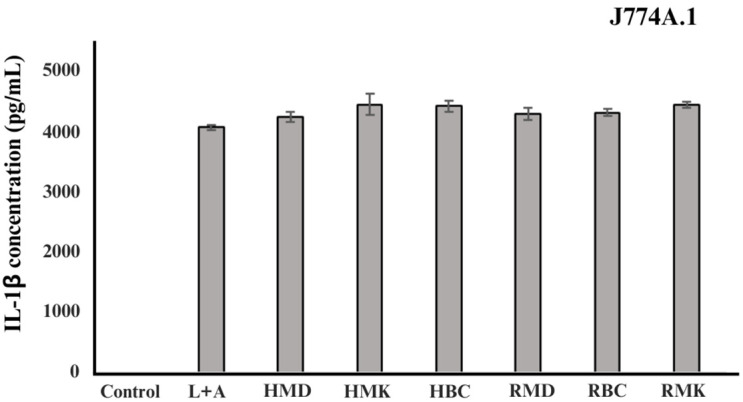
Effect of hydrosol and rose dreg fractions on the production of IL-1β in J774A.1 cells stimulated with LPS. LPS, lipopolysaccharides; ATP, adenosine triphosphate; HMD, hydrosol fractions of Mon Dang Prasert; HMK, hydrosol fractions of Mon Klai Kangwon; HBC, hydrosol fractions of Bishop’s Castle; RMD, rose dreg fractions of Mon Dang Prasert; RMK, rose dreg fractions of Mon Klai Kangwon; RBC, rose dreg fractions of Bishop’s Castle.

The inhibitory effect of H and R fractions on the production of IL-1β in J774A.1 cells stimulated with LPS is shown in Figure 3. The cells were pretreated with the sample at a concentration of 20 μg/mL, followed by stimulation with LPS. The results showed that the H and R fractions did not have an anti-inflammatory effect at this concentration. Latifi et al. [53] examined the anti-inflammatory effects of volatile oil and hydroalcoholic extract derived from *R. damascena*. on acetic acid-induced colitis in rats. Their study revealed a notable amelioration of colitis indices in rats following oral pretreatment with both extracts. This observed reduction in inflammation within the gastrointestinal tract suggested the potential therapeutic value of these plant extracts in mitigating symptoms associated with inflammatory bowel disease. Similarly, Hajhashemi et al. [54] studied the analgesic and anti-inflammatory properties of hydroalcoholic extract and essential oil from *R. damascena* in mice. Their findings revealed that the hydroalcoholic extract effectively reduced paw oedema induced by carrageenan, suggesting its potential as an anti-inflammatory agent. However, the water component of the essential oil did not exhibit any analgesic or anti-inflammatory effects in the experimental tests. Safia et al. [55] evaluated the anti-inflammatory potential of a cream formulated with the aqueous petals extract of *R. damascena*. Through their study, they utilised standard diclofenac sodium to measure the inhibition of protein denaturation, assessing the in vitro anti-inflammatory activity of various cream formulations. Notably, cream formulation F1, containing 50 g of rose water, exhibited substantial anti-inflammatory activity, registering an inhibition rate of 80.6% at a concentration of 1000 µg/mL. Maleev et al. [56] described the use of rose water as an anti-inflammatory medication. Rose water has a polyphenolic component that helps reduce inflammation. A different study by Karimi et al. [57] found that putting 3 mL of an ethanolic extract directly into the abdominal cavity of rats (at concentrations of 1% and 5%) greatly reduced fibrosis and adhesions after a laparotomy. The studies discussed provide valuable insights into the anti-inflammatory potential of extracts derived from *R. damascena*. The summary of research encompasses a broader range of experimental models, including colitis in rats, paw oedema in mice, and in vitro assays, revealing promising anti-inflammatory effects of various formulations such as hydroalcoholic extract, volatile oil, and cream formulations containing rose water. In contrast, our study focused on assessing the inhibitory effect of by-product fractions from Damask rose aromatic extraction on IL-1β production in a cellular model, with results indicating a lack of anti-inflammatory efficacy at the tested concentration. However, further investigations should elucidate the mechanisms underlying their anti-inflammatory activity and optimise their therapeutic applications.

### 2.7. Anti-Melanogenesis Activity

**Figure 4:**
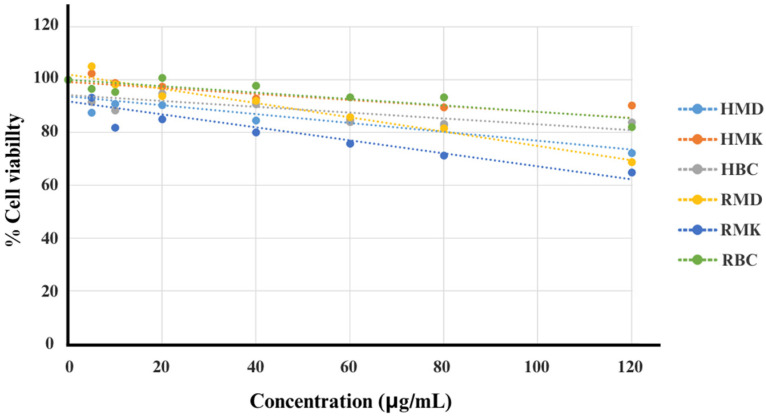
Evaluation of the cell viability (MTT assay) of hydrosol and rose dreg fractions in B16-F10 cells. HMD, hydrosol fractions of Mon Dang Prasert; HMK, hydrosol fractions of Mon Klai Kangwon; HBC, hydrosol fractions of Bishop’s Castle; RMD, rose dreg fractions of Mon Dang Prasert; RMK, rose dreg fractions of Mon Klai Kangwon; RBC, rose dreg fractions of Bishop’s Castle.

Cell viability was assessed using the MTT assay, which relies on the conversion of a yellow tetrazolium salt into a purple formazan product. The results revealed that at a concentration of 120 μg/mL, cell viability was greater than 50%, as shown in Figure 4, across all treatments compared to the control group (B16-F10 cells without treatment), suggesting that the concentrations of H and R used were not detrimental to the cells and are suitable for further assays.

**Figure 5:**
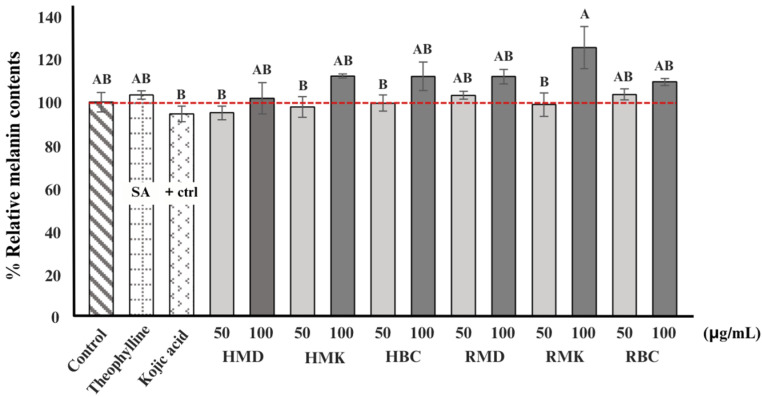
The effects of hydrosol and rose dreg fractions on melanin content in B16-F10 cells. Error bars are based on the mean ± SE of three experiments. Different letters are significantly different (*p* < 0.05) by DMRT.SA, Stimulating Agent; HMD, hydrosol fractions of Mon Dang Prasert; HMK, hydrosol fractions of Mon Klai Kangwon; HBC, hydrosol fractions of Bishop’s Castle; RMD, rose dreg fractions of Mon Dang Prasert; RMK, rose dreg fractions of Mon Klai Kangwon; RBC, rose dreg fractions of Bishop’s Castle.

Figure 5 illustrates the effects of H and R fractions on the melanin content in B16-F10 cells. B16-F10 cells were treated with theophylline to increase melanin production and kojic acid, a commercialised anti-melanogenesis chemical, to reduce melanin production. The anti-melanogenesis action of rose extracts was then examined at concentrations of 50 and 100 μg/mL. When the control produced 100% melanin, the results showed that theophylline increased melanin production by 3.35%, but kojic acid inhibited melanin formation by 5.49%. The RMK concentration of 100 μg/mL showed the greatest potential for increasing melanin production at 11.87% when compared to control blank cells. In contrast, at a concentration of 50 μg/mL, HMD, HMK, HBC, and RMK inhibited melanin formation at 4.97%, 2.26%, 0.43%, and 1.19%, respectively, when compared to control. However, the rose extract at a concentration of 50 μg/mL has a greater ability to inhibit melanin synthesis than the concentration of 100 μg/mL. Similar to the findings reported by Solimine et al. [58], rose oil distillation wastewater emerges as a by-product in the process of steam distillation of dried rose flowers for rose oil extraction. It exhibited clear inhibitory efficacy against tyrosinase, as evidenced by its IC_50_ value of 0.41  ±  0.01 μg/mL, which was nearly ten times stronger than the IC_50_ value of the positive control kojic acid. In addition, Hadipour et al. [59] evaluated the anti-melanogenic effects of Damask rose essential oil, MeOH, and various fractions, including n-hexane, dichloromethane, ethyl acetate, and n-butanol fractions, on the B16-F10 murine melanoma cell line. The results showed that the Damask rose extracts and fractions were not cytotoxic to the B16-F10 cells. Nevertheless, they exerted substantial inhibitory effects on mushroom tyrosinase activity, melanin content, and ROS production at a concentration of 200 µg/mL but not at other concentrations. In conclusion, by-product fractions from the Damask rose can slow down the production of melanin in B16-F10 mouse melanoma cells. These extracts are not cytotoxic to the cells but effectively reduce melanin content. Its antioxidant properties, which are capable of regulating melanin synthesis and acting as anti-melanogenesis agents, are responsible for its anti-melanogenic effects. This research highlights the potential of by-products as a natural ingredient for skin-whitening or anti-hyperpigmentation products, offering a safer alternative to synthetic compounds for managing skin pigmentation.

## 3. Materials and Methods

### 3.1. Chemicals

Antioxidant standards (catechin hydrate, 2,2-diphenyl-1-picrylhydrazyl, Trolox, 2,2′-azino-bis(3-ethylbenzothiazoline-6-sulfonic acid, gallic acid, 2,4,6-trypyridyl-s-triazine), standards for GCMS analysis (geraniol, (R)-(+)-Limonene), standards for HPLC analysis (gallic acid, epicatechin, caffeic acid, naringin, p-coumaric acid, rosmarinic acid, vanillic acid, o-coumaric acid, and quercetin), and analytical grade chemicals (Coomassie Brilliant blue G-250, dimethyl sulfoxide (DMSO), kojic acid, 3-(4,5-dimethylthiazol-2-yl)-2,5-diphenyltetrazolium bromide (MTT), phosphate-buffered saline (PBS), Sabouraud Dextrose Broth (SDB), theophylline, and Tryptic Soy Broth (TSB) were purchased from Sigma-Aldrich (St. Louis, MO, USA). The analytical grade chemicals (aluminium chloride, dichloromethane, ethanol, hexane, methanol, deionised water, sodium chloride, sodium bicarbonate, sodium hydroxide, sodium nitrite, sodium sulfate) and the mobile phase for HPLC (distilled water, acetonitrile, formic acid) were sourced from RCI Labscan Limited (Bangkok, Thailand). Folin–Ciocalteu reagent was purchased from Merckmillipore (Burlington, MA, USA).

### 3.2. Plant Material

In February 2023, completely bloomed fresh flowers from three varieties of organically cultivated Damask roses, specifically, Mon Dang Prasert, Mon Klai Kangwon, and Bishop’s Castle, were collected from a rose farm located in Chiang Dao District, Chiang Mai Province (latitude: 19.33133, longitude: 98.94254). The living plants were sent to Queensirikit Botanic Garden (QSBG) in Chiang Mai, Thailand, for taxonomical verification. Subsequently, they were cultivated as living specimens within the Plant Bioactive Compound Laboratory (BAC), Chiang Mai University (CMU), Chiang Mai, Thailand. The freshly harvested flowers were analysed for aroma compound using gas chromatography (GC). The remaining flowers were dried in the shade at room temperature (28 °C) until they reached an average moisture content of 5% and then underwent hydro-distillation for the volatile fraction [60].

### 3.3. Analysis of the Chemical Composition of Aroma Compounds in Three Fresh Rose Varieties at all Three Stages

The freshly picked roses of three varieties at three different stages were analysed for volatile composition using automated Solid-Phase Microextraction (SPME) similar to the methods of Mohsen et al. [61] with some modifications. Briefly, fresh flowers (200 mg) were placed in SPME screw cap vials (1.5 mL), and the SPME fibre was inserted; the vials were then placed in an oven at 50 °C for 30 min. The fibre was subsequently removed and injected into the injection port of the gas chromatography–mass spectrometer (GC-MS) (Restek, PA, USA) equipped with DB-5 column (30 m × 0.25 mm i.d. × 0.25 μm film thickness; Supelco, PA, USA) and Shimadzu QP5050A mass spectrometer. The temperatures of the interface, injector, and oven were adjusted in accordance with the experimental conditions of Issa-Issa et al. [62] using a gradient temperature programme. Helium was utilised as the carrier gas at a total flow rate of 0.9 mL/min using spitless injection mode. The quadruple mass spectrometer was operated in electron impact ionisation (EI) mode at 70 eV, and scan range was set at 40–500 m/z.

The volatile compounds detected from GC-MS were described as odour characteristics using the database from the Good Scent Company information system available at http://www.thegoodscentscompany.com/ (accessed on 11 February 2024). This research described the primary olfactory traits of roses as follows:

1. The rose scent is a unique fragrance reminiscent of rose with slightly honey sweetness and occasionally resembling the aroma of laundry powder;
2. Fruity scents typically include tropical fruit aromas, citrus fragrances, and neroli orange blossoms;
3. Green scents include fresh herbal scents, eucalyptus, pine, wood, and green vegetables;
4. Milk scents are reminiscent of powdered milk and possess similarities with the scents of starch and plant oils;
5. Fresh scents refer to the smell of fresh air that is free of any smell (odourless).

### 3.4. The Distillate Extraction of Damask Rose

Briefly, dried rose petals (30 g) were subjected to hydro-distillation using a 1 L Clevenger apparatus, which was filled with 500 mL of distilled water and extracted at 100–150 °C for approximately 1 h in a stirring heating mantle (MTopo^®^, Gyeonggi-do, South Korea) [63]. The volatiles were then isolated from the distilled part by a liquid–liquid extraction process using hexane as the extractant. The aqueous–organic ratio was constant at 10:1, and the extraction was performed in the presence of sodium chloride at a concentration of 10% *w/v* in the aqueous phase [64]. The volatile fraction (V) was separated, and the remaining liquid was labelled as the distilled fraction (D). The liquid remaining in the Clevenger apparatus was recognised as the hydrosol fraction (H), and solid was the rose dreg fraction (R).

R was then dried using a hot air oven at 70 °C for 12 h and ground into fine powder. The R powder was immersed in 70% ethanol at a ratio of 1:5 *w/v* and agitated periodically at room temperature for 48 h. The supernatant was filtrated using Whatman No.1 filter paper and evaporated to dryness [64]. However, the liquid of H and D were also evaporated using rotary evaporator (SIAM INTERCORP Co., Ltd., Bangkok, Thailand).

### 3.5. Analysis of Volatile Faction

The chemical composition of the V fraction was analysed using GC-MS. The sample was diluted in dichloromethane before being injected into a Bruker-Scion 436 GC equipped with a 30 m × 0.25 mm Rxi-5Sil MS column (Restek, PA, USA). The interface temperature was 200 °C, and mass spectra were obtained at 70 eV in EI mode, with a scanning speed of 0.5 scans/s and 20–350 m/z [65]. The process of identifying volatile components involved the comparison of mass spectra obtained from the NIST 05.L and NIST 98.L libraries.

### 3.6. Phytochemical Analysis

#### 3.6.1. Total Phenolic Content

The total phenolic content was determined using a modified method based on the procedure described by Sunanta et al. [66]. In this method, 30 µL of extracts with three replications were combined with 60 µL of Folin–Ciocalteu reagent. The mixture was then neutralised with 210 µL of 6.0% *w/v* saturated sodium bicarbonate solution. The mixture was subsequently kept at room temperature in the darkness for a duration of 2 h. The measurement of absorbance was conducted using a UV-Vis spectrophotometer (BMG LABTECH, Offenburg, Germany) at a wavelength of 725 nm. The calibration standard was generated by using a range of gallic acid concentrations (10–200 mg/mL). The total phenolic content was expressed as mg of gallic acid equivalents (GAE) per g of dried sample.

#### 3.6.2. Total Flavonoid Content

A measurement of total flavonoid concentration was carried out using a modified methodology developed by Khamsaw et al. [67]. The extracts (25 µL) with three replications were mixed with 125 µL of distilled water and 7.5 µL of 5.0% NaNO_2_ solution. The combination was allowed to stand at room temperature for 5 min. Subsequently, a volume of 15 µL of 10.0% AlCl_3_·6H_2_O was added to the mixture and incubated for a period of 6 min. After that, a volume of 50 µL of 1 M of NaOH and 27.5 µL distilled water was added. The absorbance of the mixture was measured at a wavelength of 510 nm using the UV-Vis spectrophotometer. The catechin calibration standard was generated using various concentration ranges (30–300 mg/mL). The total flavonoid content was expressed as mg catechin equivalents (CE) per g of dried sample.

#### 3.6.3. Antioxidant Activities

##### Determination of 2,2-Diphenyl-1-Picrylhydrazyl (DPPH) Radical Scavenging Activity

The methodology that was applied to assess the free radical scavenging activity was based on the protocol outlined by Sunanta et al. [68]. A volume of 25 µL of the extracts with three replications was combined with 250 µL of 0.2 mM DPPH (2,2-diphenyl-1-picrylhydrazyl). The mixture was then incubated in the darkness at room temperature for 30 min. The absorbance was measured at a specific wavelength of 510 nm using the UV-Vis spectrophotometer. The calculation of DPPH radical scavenging was carried out using the following Equation (1):

(1) $$DPPH\;\mathrm{radical}\;\mathrm{scavenging}\;\mathrm{activity}\;=\frac{{Abs}_{control}-{Abs}_{sample}}{{Abs}_{control}}\times 100$$

where Abs_control_ is the absorbance of DPPH radical mixed with 80% methanol, and Abs_sample_ is the absorbance of DPPH radical reacted with sample extract

The antioxidant capacities of samples were measured by comparing them to a Trolox standard and expressed as mg Trolox equivalents (TE) per g of dried sample. The IC_50_ value represents the concentration of the sample required to block 50% of DPPH free radicals during the process of scavenging. The IC_50_ value was established by graphically analysing the curve plot that shows the relationship between the percentages of DPPH scavenging activity and the concentration of the sample.

##### Determination of 2,2′-Azino-Bis (3-Ethylbenzothiazoline-6-Sulfonic Acid) (ABTS) Radical Scavenging Activity

The ABTS [2,2-azino-bis-(3-ethylbenzothiazoline-6-sulfonic acid)] assay was described by Sangta et al. [69]. Firstly, the working solution was prepared by mixing the two stock solutions, including 7.0 mM ABTS solution and 2.45 mM potassium persulfate solution. The solutions were combined in equal proportions, and the solution was allowed to react in the darkness at room temperature for 12–16 h. The ABTS solution was prepared by diluting 1.0 mL ABTS with 60.0 mL of 80.0% methanol in order to get an absorbance of 0.7 ± 0.02 units at a wavelength of 734 nm. Subsequently, 10 µL of the extract and 200 µL of ABTS working solution were transferred into the 96-well plate; the solution was then shaken and incubated at room temperature for 30 min. The absorbance was measured at 734 nm. The analyses were done by three replications. The ABTS scavenging capacity of the extract was calculated using the following Equation (2):

(2) $$ABTS\;\mathrm{radical}\;\mathrm{scavenging}\;\mathrm{activity}\;(\%)=\frac{{Abs}_{control}-{Abs}_{sample}}{{Abs}_{control}}\times 100$$

where Abs_control_ is the absorbance of ABTS radical mixed with 80% methanol, and Abs_sample_ is the absorbance of ABTS radical reacted with sample extract.

By comparing samples to a Trolox standard, their antioxidant capacities were determined and expressed in mg TE per g of the sample. The IC_50_ value represents the concentration of the sample required to block 50% of ABTS free radicals during the process of scavenging. The IC_50_ value was established by graphically analysing the curve plot that shows the relationship between the percentages of ABTS scavenging activity and the concentration of the sample.

##### Determination of Ferric Reducing Antioxidant Power (FRAP)

The ferric reducing antioxidant power assay was conducted using the methodology outlined by Prasad et al. [70]. The FRAP reagent was prepared by combining 2.5 mL of heated 10 mM 2,4,6-trypyridyl-s-triazine, 2.5 mL of 20 mM FeCl_3_ solution, and 25 mL of 300 mM acetate buffer at a pH of 3.6. Prior to use, the reagent combination was preheated to a temperature of 37 °C. A volume of 150 μL of FRAP reagent was incubated with the extracts (20 μL). Additionally, Trolox (25–200 mg/mL) was used as a standard. The incubation was carried out in darkness for a duration of 30 min, after which the absorbance of the resultant blue colour was measured at a wavelength of 593 nm. Three replicates were utilised to conduct the analysis. The FRAP value of the extract was calculated using the following Equation (3):

(3) $$\mathrm{FRAP}\;\mathrm{value}\;=\frac{{(A}_{1}-A_{0})}{{(A}_{c}-A_{0})}\times 100$$

where A_C_ is the absorbance of the positive control.

A_1_ is the absorbance of the sample.
A_0_ is the absorbance of the blank.

The antioxidant capacities of the samples were quantified in mg TE per g of dehydrated sample by comparing them to a Trolox standard. The IC_50_ value represents the concentration of the sample required to block 50% of FRAP free radicals during the process of scavenging. The IC_50_ value was established by graphically analysing the curve plot that shows the relationship between the percentages of FRAP scavenging activity and the concentration of the sample.

### 3.7. Quantitative Analysis of Phenolic and Flavonoid

The quantification of phenolic and flavonoid contents in H and R fractions was carried out utilising HPLC, following the modified protocols by Sangta, Wongkaew, Tangpao, Withee, Haituk, Arjin, Sringarm, Hongsibsong, Sutan, and Pusadee [69]. The analysis for polyphenol contents was conducted using an HPLC system (Shimadzu, Kyoto, Japan) equipped with an automatic injection module (SIL-20ACHT) and diode array detection (CTO-20AC). Reverse-phase column chromatography was performed with an Ultra Aqueous C18 column (Poroshell 120 EC-C18 4.6 × 100 mm, 2.7 2.5 µm) (RESTEK, PA, USA). The mobile phase consisted of a mixture of A and B, where A contained formic acid and distilled water in a 1:99 ratio, and B included acetonitrile (ACN), formic acid, and distilled water in an 85:50:1 ratio with a flow rate of 1 mL/min and an injection volume set at 10 μL. The gradient elution began with 80% A for 4 min, then decreased to 25% over 8 min. The elution was maintained for 2 min, then shifted to 70% A in 3 min, and then returned to 95% A in 1 min. The total run time was 18 min, and all determinations were performed in triplicate. Chromatograms were recorded using photodiode array detection at 280 nm. Calibration standards were prepared through serial dilutions of different polyphenols (gallic acid, epicatechin, caffeic acid, naringin, p-coumaric acid, rosmarinic acid, vanillic acid, o-coumaric acid, and quercetin) to achieve concentrations ranging from 50 to 500 μg/mL.

### 3.8. Antimicrobial Activity

A total of six commonly used microbial strains were used in order to evaluate the antibacterial properties of H and R fractions. These strains consisted of three Gram-positive bacteria (*Staphylococcus epidermidis*; *S. epidermidis* ATCC12228, *Staphylococcus aureus*; *S. aureus* ATCC25923, and *Cutibacterium acnes*; C. acnes (formerly *Proprionibacterium acnes*) ATCC6919), two Gram-negative bacteria (*Escherichia coli*; *E. coli* ATCC25922 and *Pseudomonas aeruginosa*; *P. aeruginosa* ATCC27853), and a yeast (*Candida albicans*; *C. albicans* ATCC90028). The microorganisms were obtained from the culture collection of Microbiological Laboratory, Faculty of Pharmacy, CMU. The plates inoculated with the bacteria and fungi cultures were incubated overnight at 37 °C.

The determination of the Minimum Inhibitory Concentration (MIC), Minimum Bactericidal Concentration (MBC), and Minimum Fungicidal Concentration (MFC) was carried out using the method of Ghavam [13] and Butrungrod et al. [71] with slight modification. The Tryptic Soy Broth (TSB) for bacteria and Sabouraud Dextrose Broth (SDB) for yeast medium were added in sterilised microplate well; then, H and R extracts were added at a concentration of 20 mg/mL for 100 µL into the first and second columns and subsequently underwent a two-fold serial dilution across the entirety of the plate. Following this, a total of 100 µL of bacteria or fungus was added to each individual well. The plates containing bacterial and fungal cultures were subjected to incubation at a temperature of 37 °C for a duration of 48 h. The determination of MIC involved assessing the turbidity (compared to MaFarland’s standard No. 0.5 at the concentration of bacteria at 1 × 10^8^ and yeast at 1 × 10^6^ CFU/mL) observed in each well of the microplate. The experiment was performed in triplicate for each sample, and the mean value was documented as the MIC of the samples that demonstrated inhibitory effects on the growth of bacteria or yeast. Inhibitory concentrations were cultured on Tryptic Soy Agar (TSA) and Sabouraud Dextrose Agar (SDA) for bacteria and yeast, respectively, to determine the MBC or MFC.

### 3.9. Anti-Inflammatory

J774A.1 murine macrophage cells were obtained from American Type Culture Collection (ATCC) and cultured in Roswell Park Memorial Institute (RPMI) 1640 medium containing 10% heat-inactivated foetal bovine serum (Hyclone Co., Ltd., Logan, UT, USA), along with 2 mM glutamine (Life Technologies, Inc., Carlsbad, MD, USA). Subsequently, the cells were maintained in incubation conditions at 37 °C with 5% CO_2_. To investigate the inhibitory effect of H and R fractions on the production of IL-1β in J774A.1 cells stimulated with lipopolysaccharides (LPS), the cells were pretreated using the method of Chao et al. [72] and Hua et al. [73] with the sample at a concentration of 20 μg/mL for 30 min at 37 °C, followed by stimulation with LPS (1 μg/mL) for 4 h. Subsequently, adenosine triphosphate (ATP) was introduced to the cells at a concentration of 200 mM for 30 min. Insoluble material in the cell culture supernatant was then removed via centrifugation at 4 °C for 15 min at 12,000 g. The MRX microplate reader (Dynex Technologies, Chantilly, VA, USA) was utilised to measure the absorbance of the samples at a wavelength of 450 nm.

### 3.10. Anti-Melanogenesis Activity

#### 3.10.1. Cell Culture

B16-F10 murine melanoma cells were obtained from ATCC and cultured in Dulbecco’s Modified Eagle Medium (DMEM) supplemented with 10% FBS, 1% penicillin/streptomycin (Hyclone Co., Ltd., Logan, UT, USA), along with 1% L-glutamine (Thermo Fisher Scientific, Carlsbad, CA, USA). Subsequently, the cells were maintained in incubation conditions at 37 °C with 5% CO_2_.

#### 3.10.2. MTT Assay for Cell Viability

Cell viability of H and R fractions was determined using an MTT assay as described by Hsu et al. [74] with minor changes. Briefly, B16-F10 cells were seeded in a 96-well plate (5000 cells per well) and then incubated for 24 h. Before use, the samples were dissolved in an equivalent volume of deionised water. The cells were treated with or without the extract (0, 5, 10, 20, 40, 60, 80, and 120 µg/mL) for 48 h. After additional incubation, the medium was aspirated, and 15 μL of MTT solution (5 mg/mL in PBS) was added to each well. The cells were incubated at 37 °C for 4 h. Subsequently, 200 μL of DMSO was used to dissolve the crystals in each well. The measurement of optical density was conducted at a wavelength of 570 nm.

#### 3.10.3. Melanin Content Assay

Melanin content was measured as described previously by Chan et al. [75] with slight modifications. The B16-F10 melanoma cells were harvested using trypsin-EDTA (Thermo Fisher Scientific, CA, USA) and subsequently seeded at a density of 100,000 cells per culture dish in 5 mL of medium. The cells were then incubated at 37 °C in a 5% CO_2_ atmosphere for 24 h, allowing for overnight adhesion. Following this, the cells were treated with theophylline at 100 μM, and the melanin content was determined after treatment with the H and R fractions at concentrations of 50 and 100 μg/mL, respectively, as well as kojic acid at a concentration of 200 μM, which served as the positive control for whitening property. This treatment lasted for 48 h. Subsequently, the cells were washed with PBS, followed by lysis using 200 μL of 1 N NaOH, and incubated at 80 °C for 1 h. The absorbance of the cell lysate was then measured at 490 nm.

### 3.11. Statistical Analysis

The experiments were performed with a minimum of three replicates. The mean differences among all fractions were evaluated using a one-way analysis of variance and Duncan’s multiple range test. The statistical analyses were conducted using the SPSS 23.0 software (SPSS Inc., Chicago, IL, USA). A *p*-value at < 0.05 was deemed to be statistically significant. This research used principal component analysis (PCA) in XLSTAT version 2020 to examine the relationships between polyphenol compositions, antioxidants, and antibacterials.

## 4. Conclusions

This study identified phenylethyl alcohol as the key contributor to the characteristic sweet rosy aroma of fresh Damask rose flowers and their volatile fractions across the three investigated varieties. To maximise the value of by-products generated during volatile oil extraction, the distillate, hydrosol, and rose dregs were further analysed for potential pharmaceutical applications. Interestingly, the hydrosol exhibited the highest concentration of total phenolics and flavonoids, demonstrating significant antioxidant activity and the capacity to reduce melanogenesis. Conversely, the by-products lacked significant antimicrobial or anti-inflammatory properties. The observed antioxidant activity renders these by-products promising candidates for utilisation as intermediate materials in the development of cosmetic and pharmaceutical formulations. This approach not only promotes the valorisation of agricultural by-products and minimises environmental concerns but also signifies a noteworthy contribution towards achieving sustainable development goals within the Thai agro-industry.

## 5. Patents

This section is not mandatory but may be added if there are patents resulting from the work reported in this manuscript.

## Figures and Tables

**Table 1:** Morphology and aroma characteristics of three fresh rose species.

Sample	Scientific Name, Morphology, and Basic Odour Characteristics	Moisture Content (%)	Scent Intensity		
			Bud Stage 1	Initial Opening Stage 2	Full Bloom Stage 3
Mon Dang Prasert 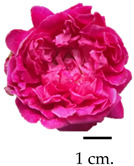	Family: Rosaceae Species name: *Rosa damascena* Shrubs, stems, and thorny branches. The leaves are composed of feathers, arranged alternately; the leaves are oval-shaped, rounded, and pointed at the tip; the edge of the leaf is serrated like a saw-tooth. The diameter is 2.5–3.5 cm. The flowers are clustered into clusters of 3–5 flowers at the tip of the top. With dark red petals when exposed to sunlight and pink petal base, the petals are stacked in several layers. When the flowers are in full bloom, they are 5–7 cm. in diameter and have a mild fragrance.	86.34	+	++	++
Mon Klai Kangwon 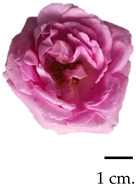	Family: Rosaceae Species name: *Rosa damascena* Shrubs, stems, and branches have thorns. Characteristics of feathered compound leaves arranged alternately and short-paired leaves. The leaves are oval-shaped, 2–3 cm in diameter, with a rounded, pointed tip and a saw-tooth serrated edge. The flowers are clustered into clusters of 3–5 flowers at the tip of the top. The light-coloured pinkish petals are neatly stacked in several layers. When the flowers are in full bloom, they are 5–7 cm. in diameter and have a strong fragrance.	87.37	++	+++	+++
Bishop’s Castle 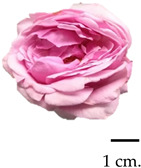	Family: Rosaceae Species name: *Rosa ‘Ausbecks’* Shrubs, stems, and thorny branches. The leaves are compound, feathery, alternate, oval-shaped, 3–5 cm in diameter, rounded at the base, pointed at the edge, serrated, and saw-toothed. The flowers are clustered into clusters of 2–3 flowers at the tip of the top. The petals are light pink and stacked tightly in several layers. When the flowers are in full bloom, they are about 5–7 cm. in diameter and have a powdery fragrance.	86.04	+	++	+++

The symbol “+” represents a scent intensity level, with “++” being the lowest intensity and “+++” representing the maximum intensity.

**Table 2:** Bioactive compounds and antioxidant activity of by-product fractions from Damask rose.

Samples	Flavonoids (mg catechin/g Dried Sample)	Phenolics (mg gallic/g Dried Sample)	ABTS			DPPH			FRAP		
			% Inhibition	IC_50_ (μg/mL)	TEAC (mg/g)	% Inhibition	IC_50_ (μg/mL)	TEAC (mg/g)	% Inhibition	IC_50_ (μg/mL)	TEAC (mg/g)
HMD	6.93 ± 0.41 ^Aa^	10.08 ± 0.55 ^Aa^	58.87 ± 1.67 ^Ba^	0.97 ± 0.03 ^Dc^	0.12 ± 0.00 ^Aa^	55.91 ± 1.75 ^ABa^	0.95 ± 0.02 ^Cc^	0.12 ± 0.00 ^Aa^	52.49 ± 0.36 ^Ba^	0.67 ± 0.01 ^Dc^	3.89 ± 0.11 ^Aa^
HMK	4.61 ± 0.17 ^Bb^	10.56 ± 0.71 ^Aa^	52.85 ± 0.85 ^Cb^	1.10 ± 0.01 ^CDb^	0.11 ± 0.00 ^Bb^	50.95 ± 0.46 ^BCb^	1.03 ± 0.00 ^Cb^	0.11 ± 0.00 ^Bb^	46.60 ± 0.43 ^Cb^	0.73 ± 0.01 ^CDb^	3.45 ± 0.28 ^Bb^
HMB	4.39 ± 0.12 ^Bb^	8.14 ± 0.62 ^Bb^	43.85 ± 1.80 ^Dc^	1.36 ± 0.06 ^Ca^	0.09 ± 0.00 ^Cc^	45.25 ± 1.62 ^Cc^	1.17 ± 0.04 ^Ca^	0.10 ± 0.00 ^Cc^	41.48 ± 0.86 ^Cc^	0.81 ± 0.02 ^Ca^	3.15 ± 0.47 ^Cc^
RMD	3.22 ± 0.24 ^Ca^	5.89 ± 0.46 ^Ca^	65.80 ± 3.47 ^Aa^	2.22 ± 0.12 ^Bb^	0.05 ± 0.00 ^Da^	58.03 ± 2.46 ^ABa^	2.31 ± 0.09 ^Bb^	0.05 ± 0.00 ^Da^	54.22 ± 1.85 ^Aba^	1.67 ± 0.04 ^Bb^	1.62 ± 0.66 ^Da^
RMK	2.23 ± 0.08 ^Db^	5.13 ± 0.19 ^Ca^	57.90 ± 1.60 ^BCb^	1.97 ± 0.21 ^Bb^	0.05 ± 0.00 ^Db^	60.85 ± 4.75 ^Aa^	2.15 ± 0.14 ^Bb^	0.05 ± 0.00 ^Da^	58.24 ± 3.40 ^Aa^	1.63 ± 0.08 ^Bb^	1.82 ± 0.15 ^Da^
RMB	1.58 ± 0.09 ^Ec^	3.75 ± 0.14 ^Db^	44.24 ± 0.75 ^Dc^	3.32 ± 0.06 ^Aa^	0.04 ± 0.00 ^Ec^	44.63 ± 0.55 ^Cb^	2.80 ± 0.03 ^Aa^	0.04 ± 0.00 ^Eb^	42.88 ± 0.64 ^Cb^	2.03 ± 0.09 ^Aa^	1.29 ± 0.01 ^Eb^
DMD	0.00 ± 0.00 ^Fa^	0.00 ± 0.00 ^Ea^	18.77 ± 0.35 ^Fb^	n/a	0.02 ± 0.01 ^Fa^	8.28 ± 2.81 ^Da^	n/a	0.01 ± 0.00 ^Fa^	4.40 ± 0.58 ^Fb^	n/a	0.79 ± 0.01 ^FGb^
DMK	0.00 ± 0.00 ^Fa^	0.00 ± 0.00 ^Ea^	17.81 ± 3.55 ^Fb^	n/a	0.00 ± 0.00 ^Gb^	5.29 ± 3.49 ^Dab^	n/a	0.01 ± 0.00 ^Fa^	6.47 ± 1.16 ^Fb^	n/a	0.87 ± 0.15 ^Fa^
DMB	0.00 ± 0.00 ^Fa^	0.00 ± 0.00 ^Ea^	30.88 ± 2.28 ^Ea^	n/a	0.01 ± 0.01 F^Gab^	0.13 ± 0.76 ^Db^	n/a	0.00 ± 0.00 ^Fb^	14.95 ± 1.46 ^Ea^	n/a	0.87 ± 0.01 ^Fa^
VMD	0.06 ± 0.00 ^Fa^	0.02 ± 0.02 ^Ea^	21.94 ± 0.48 ^Fa^	n/a	0.00 ± 0.00 ^Ga^	3.81 ± 0.06 ^Da^	n/a	0.01 ± 0.00 ^Fa^	14.85 ± 0.85 ^Eb^	n/a	0.86 ± 0.01 ^Fb^
VMK	0.05 ± 0.01 ^Fa^	0.01 ± 0.01 ^Ea^	4.50 ± 0.24 ^Gc^	n/a	0.00 f ± 0.00 ^Ga^	2.48 ± 0.72 ^Da^	n/a	0.01 ± 0.00 ^Fa^	26.23 ± 3.65 ^Da^	n/a	1.00 ± 0.05 ^Fa^
VMB	0.05 ± 0.01 ^Fa^	0.01 ± 0.00 ^Ea^	8.29 ± 0.57 ^Gb^	n/a	0.00 ± 0.00 ^Ga^	8.26 ± 6.66 ^Da^	n/a	0.01 ± 0.01 ^Fa^	24.60 ± 2.40 ^Da^	n/a	0.98 ± 0.03 ^Fab^

n/a = no activity; DPPH, 2-diphenyl-1-picrylhydrazyl; ABTS, 2,2-azino-bis (3-ethylbenzothiazoline-6-sulfonic acid); FRAP, ferric reducing antioxidant power; TEAC, Trolox equivalent antioxidant capacity; HMD, hydrosol fractions of Mon Dang Prasert; HMK, hydrosol fractions of Mon Klai Kangwon; HBC, hydrosol fractions of Bishop’s Castle; RMD, rose dreg fractions of Mon Dang Prasert; RMK, rose dreg fractions of Mon Klai Kangwon; RBC, rose dreg fractions of Bishop’s Castle; DMD, distillate fractions of Mon Dang Prasert; DMK, distillate fractions of Mon Klai Kangwon; DBC, distillate fractions of Bishop’s Castle; VMD, volatile fractions of Mon Dang Prasert; VMK, volatile fractions of Mon Klai Kangwon; VBC, volatile fractions of Bishop’s Castle. Values are mean of the triplicates ± SE; values followed by different letters in the same row are significantly different (*p* < 0.05) by Duncan’s multiple range test (DMRT).

**Table 3:** Identification of phenolic compounds by high-performance liquid chromatography analysis (HPLC) of hydrosol and rose dreg fractions.

Sample (mg/g Dried Sample)	HMD	HMK	HBC	RMD	RMK	RBC
Gallic acid	8.71 ± 0.09 ^E^	14.17 ± 0.03 ^A^	10.68 ± 0.11 ^D^	11.65 ± 0.16 ^C^	12.84 ± 0.07 ^B^	8.08 ± 0.03 ^F^
Epicatechin	nd	nd	nd	nd	nd	nd
Catechin/ caffeic acid	1.93 ± 0.03 ^F^	7.83 ± 0.06 ^A^	3.54 ± 0.06 ^D^	3.56 ± 0.05 ^C^	5.19 ± 0.02 ^B^	3.09 ± 0.01 ^E^
Naringin	2.92 ± 0.02 ^B^	2.71 ± 0.02 ^C^	3.38 ± 0.02 ^A^	2.50 ± 0.03 ^D^	1.78 ± 0.02 ^E^	2.53 ± 0.02 ^D^
p-Coumaric acid	7.38 ± 0.02 ^A^	6.48 ± 0.04 ^B^	nd	nd	4.35 ± 0.02 ^C^	nd
Rosmarinic acid	3.45 ± 0.01 ^B^	6.58 ± 0.01 ^A^	2.92 ± 0.02 ^C^	2.66 ± 0.02 ^D^	2.04 ± 0.02 ^E^	1.92 ± 0.04 ^F^
Vanillic acid	1.57 ± 0.01 ^C^	1.68 ± 0.00 ^B^	4.07 ± 0.02 ^A^	1.27 ± 0.01 ^E^	1.37 ± 0.01 ^D^	0.87 ± 0.01 ^F^
Quercetin	nd	nd	nd	nd	2.71 ± 0.02 ^B^	3.77 ± 0.03 ^A^
o-Coumaric acid	nd	nd	nd	nd	nd	nd

nd = not detected; HMD, hydrosol fractions of Mon Dang Prasert; HMK, hydrosol fractions of Mon Klai Kangwon; HBC, hydrosol fractions of Bishop’s Castle; RMD, rose dreg fractions of Mon Dang Prasert; RMK, rose dreg fractions of Mon Klai Kangwon; RBC, rose dreg fractions of Bishop’s Castle; Values are mean of the triplicates ± SE; values followed by different letter in the same row are significantly different (*p* < 0.05) by DMRT.

**Table 4:** Antibacterial activity of hydrosol and rose dreg fractions.

Tests	*S. aureus*	*C. acnes*	*S. epidermidis*	*C. albicans*	*P. aeruginosa*	*E. coli*
HMD	n/a *	n/a	n/a	n/a	n/a	n/a
HMK	n/a	n/a	n/a	n/a	n/a	n/a
HMB	n/a	n/a	n/a	n/a	n/a	n/a
RMD	n/a	n/a	n/a	n/a	n/a	n/a
RMK	n/a	n/a	n/a	n/a	n/a	n/a
RMB	n/a	n/a	n/a	n/a	n/a	n/a
Clinda M	≥1:2 **	≥1:512	≥1:2	n/a	n/a	n/a
Penicillin G (µg/mL)	0. 625	0.078	0.156	n/a	n/a	n/a

* n/a = no activity. ** Titer 2 means that the test has a concentration of antibiotic agent that can act against the microbial strains at a dilution of 1:2. The higher the titer, the greater the concentration of antimicrobial activity. HMD, hydrosol fractions of Mon Dang Prasert; HMK, hydrosol fractions of Mon Klai Kangwon; HBC, hydrosol fractions of Bishop’s Castle; RMD, rose dreg fractions of Mon Dang Prasert; RMK, rose dreg fractions of Mon Klai Kangwon; RBC, rose dreg fractions of Bishop’s Castle.

## Data Availability

Data will be made available on request.

## Supplementary Materials

The following supporting information can be downloaded at https://www.mdpi.com/article/10.3390/plants13121605/s1. Table S1: Chemical composition of aroma compounds of fresh roses from three varieties at three stages of budding (S1), starting to blooming (S2), and full bloom (S3); Table S2: Chemical composition of aroma compounds in volatile fractions of three rose varieties.
