# Supplementary material for: Pharmaceutical Potential Evaluation of Damask Rose By-Products from Volatile Oil Extraction
Source: Plants (Basel). 2024 Jun 9;13(12):1605. doi: 10.3390/plants13121605 (PMC11207781; doi:10.3390/plants13121605)
Supplement: Supplementary file 1 [file plants-13-01605-s001.zip › plants-3043617-supplementary.pdf]

Table S1. Chemical composition of aroma compounds of fresh roses from three varieties at three stages of bud stage (S1), start blooming (S2), and full bloom (S3).

No	Chemical name	CAS number	RI	Odour description	MD (%)			MK (%)			BC (%)		
					S1	S2	S3	S1	S2	S3	S1	S2	S3
1	2-(dimethylamino ethyl) ester Acetic acid			ether sweet fruity	nd	nd	23.61	nd	nd	23.61	nd	nd	nd
2	α-Pinene	7785-26-4	969.68	woody pine, terpene, camphor, herbal, spicy, tropical	10.61	5.92	nd	nd	nd	nd	nd	nd	10.61
3	(-)-β-Pinene	18172-67-3	1035.44	cooling, woody, piney, and turpentine-like with a fresh minty, eucalyptus, and camphoraceous note with a spicy, peppery, and nutmeg nuance.	nd	12.60	nd	nd	nd	nd	nd	nd	nd
4	5-Hepten-1-ol,2-ethenyl-6-methyl-		1037.32	fruity, apple, musty, ketonic, and creamy, with slight cheesy and banana nuances	nd	nd	nd	1.50	nd	nd	1.50	nd	nd

No	Chemical name	CAS number	RI	Odour description	MD (%)			MK (%)			BC (%)		
					S1	S2	S3	S1	S2	S3	S1	S2	S3
5	4-Hexen-1-ol, acetate	72237-36-6	1040.10	very powerful, pungent, green vegetable, and oily	nd	nd	nd	nd	nd	nd	nd	nd	nd
6	(R)-1-methyl-5-(1-methylethenyl) Cyclohexene	1461-27-4	1061.39	n/d	nd	nd	nd	0.76	nd	nd	0.76	nd	nd
7	N-carbobenzyloxy-l-tyrosyl-l-valine		1067.57	n/d	nd	nd	nd	nd	0.80	nd	nd	0.80	nd
8	Undecane	1120-21-4	1130.69	herbal, eucalyptus, and woody	nd	30.43	nd	nd	nd	nd	nd	nd	nd
9	Farnesane	502-61-4	1131.45	citrus herbs, lavender, bergamot, myrrh, neroli, green	3.79	nd	nd	nd	nd	nd	nd	nd	3.79
10	Phenylethyl Alcohol	60-12-8	1138.26	rose, sweet, floral, fresh, bread, and honey lend an intense green top note to heavier fragrances.	nd	nd	nd	57.24	nd	nd	57.24	nd	nd

No	Chemical name	CAS number	RI	Odour description	MD (%)			MK (%)			BC (%)		
					S1	S2	S3	S1	S2	S3	S1	S2	S3
11	7-methyl-3-methylene-6-Octen-1-ol	13066-51-8	1265.89	n/d	nd	nd	nd	26.92	nd	nd	26.92	nd	nd
12	Citronellol	106-22-9	1280.31	floral, leather, waxy, rose, bud, citrus	nd	nd	nd	nd	nd	nd	nd	nd	nd
13	Acetic acid, 2-phenylethyl ester	103-45-7	1309.09	floral rose, sweet honey, fruity tropical	nd	nd	nd	nd	nd	nd	nd	nd	nd
14	Citral	5392-40-5	1331.08	sharp, lemon, sweet	nd	nd	nd	nd	5.21	nd	nd	5.21	nd
15	1-Oxaspiro [4.5] dec-6-ene, 2,6,10,10-tetramethyl-	36431-72-8	1374.15	fruity, woody, cooling, minty, and camphoreous, with a fresh green herbal nuance	15.04	nd	nd	nd	40.19	nd	nd	40.19	15.04
16	Decyl vinyl ether	765-05-9	1406.44	clean, waxy, rose, fresh laundry, powdery	nd	2.19	nd	nd	nd	nd	nd	nd	nd
17	Z, Z, Z-1,4,6,9-Nonadecatetraene		1420.07	n/d	nd	3.39	nd	nd	nd	nd	nd	nd	nd

No	Chemical name	CAS number	RI	Odour description	MD (%)			MK (%)			BC (%)		
					**S1**	**S2**	**S3**	**S1**	**S2**	**S3**	**S1**	**S2**	**S3**
18	2,6-Dimethyl 2,6-octadiene	2792-39-4	1451.73	n/d	nd	nd	nd	nd	nd	nd	nd	nd	nd
19	Geranyl vinyl ether	17957-93-6	1457.41	fruity	nd	nd	nd	0.86	42.21	nd	0.86	42.21	nd
20	2,6-Dimethyl-2,6-octadien-8-yl acetate	141-12-8	1488.52	rose, green odor with lavender undertones	nd	nd	n/d	4.71	nd	nd	4.71	nd	nd
21	β-Elemene	515-13-9	1500.92	herbal, waxy, fresh	nd	nd	nd	nd	nd	nd	nd	nd	nd
22	β-Copaene-4α-ol	126060-41-1	1529.50	woody, spicy, honey	nd	23.89	nd	nd	nd	nd	nd	nd	nd
23	Caryophyllene	87-44-5	1530.22	clove, pepper, spicy, woody, dry, and elegant	nd	nd	18.90	nd	nd	18.90	nd	nd	nd
24	10,10-Dimethyl-2,6-dimethylenebicyclo[7.2.0]undecane	357414-37-0	1538.52	n/d	nd	nd	2.24	nd	nd	2.24	nd	nd	nd
25	Tricyclo[4.4.0.02,7]decan-4-ol	124753-76-0	1539.70	n/d	40.09	nd	nd	nd	nd	nd	nd	nd	40.09
26	Isogermacrene D	317819-80-0	1560.31	woody earthy spicy	nd	nd	nd	nd	nd	nd	nd	nd	nd
27	Humulene	6753-98-6	1563.95	woody, oceanic-watery, and spicy-clove	nd	nd	0.47	nd	nd	0.47	nd	nd	nd

No	Chemical name	CAS number	RI	Odour description	MD (%)			MK (%)			BC (%)		
					S1	S2	S3	S1	S2	S3	S1	S2	S3
28	α-Amorphene	483-75-0	1583.16	fruity	nd	nd	nd	0.50	nd	nd	0.50	nd	nd
29	Germacrene D	23986-74-5	1588.64	woody, earthy, spicy	nd	nd	nd	nd	nd	nd	nd	nd	nd
30	E-2-Hexadecacen-1-ol		1590.82	fresh green, leafy, fruity, unripe banana	nd	nd	1.67	nd	nd	1.67	nd	nd	nd
31	Phenethyl isovalerate	140-26-1	1596.67	floral	nd	nd	nd	nd	2.40	nd	nd	2.40	nd
32	Alloaromadendrene	25246-27-9	1605.85	n/d	0.85	nd	nd	nd	nd	nd	nd	nd	0.85
33	Hexadecane	544-76-3	1607.69	odorless,	nd	nd	0.40	nd	nd	0.40	nd	nd	nd
34	α-Farnesene	502-61-4	1609.16	citrus, herbal, lavender, bergamot, myrrh, neroli, green	nd	nd	nd	nd	nd	nd	nd	nd	nd
35	β-Cubebene	13744-15-5	1612.46	citrus, fruity, radish	1.20	1.57	nd	nd	nd	nd	nd	nd	1.20
36	Cadina-1(6),4-diene	20085-11-4	1617.91	n/d	nd	nd	nd	nd	nd	nd	nd	nd	nd
37	8-Heptadecene	16369-12-3	1720.27	n/d	nd	nd	0.84	nd	nd	0.84	nd	nd	nd
38	Benzoic acid, 2-phenylethyl ester	94-47-3	1821.36	rose, balsamic, honey, floral	nd	nd	n/d	nd	0.34	nd	nd	0.34	nd
39	9-Nonadecene	31035-07-1	1858.06	fatty	15.71	8.61	26.75	3.27	4.58	26.75	3.27	4.58	15.71

No	Chemical name	CAS number	RI	Odour description	MD (%)			MK (%)			BC (%)		
					S1	S2	S3	S1	S2	S3	S1	S2	S3
40	Eicosane	112-95-8	1892.86	primarily used in rose and tuberose complexes	10.17	9.24	23.05	4.24	2.52	23.05	4.24	2.52	10.17
41	11,14-Eicosadienoic acid	2091-39-6	1952.91	milky	0.59	nd	2.08	nd	nd	2.08	nd	nd	0.59
42	9,17-Octadecadienal, (Z)-	56554-35-9	1954.13	n/d	nd	nd	nd	nd	0.36	nd	nd	0.36	nd
43	Tetracosane	646-31-1		floral	1.94	2.16	nd	nd	1.39	nd	nd	1.39	1.94

CAS = Chemical Abstracts Service; RI = Retention index; nd = not detected; n/d = no data; Mon Dang Prasert (MD), Mon Klai Kangwon (MK); Bishop's Castle (BC)

Table S2. Chemical composition of aroma compounds in volatile fractions of three rose varieties.

No	Chemical name	RI	MD (%)	MK (%)	BC (%)
1	Furfural	861.7	24.92	12.05	24.92
2	2-Furanmethanol	885.29	0.34	nd	0.34
3	Butanoic acid	910.05	0.06	nd	0.06
4	Heptanal	935.71	nd	0.65	nd
5	5-methyl-2-Furancarboxaldehyde	993.56	1.74	nd	1.74
6	Benzaldehyde	994.6	nd	0.93	nd
7	β-Cymene	1053.94	0.45	0.42	0.45
8	Benzyl alcohol	1061.59	0.78	1.91	0.78
9	Benzeneacetaldehyde	1069.8	1.40	1.12	1.40
10	Decane, 2,4,6-trimethyl-	1080.98	0.67	0.33	0.67
11	4-Carene, (1S,3R,6R)-(-)-	1082.88	0.28	nd	0.28
12	Bicyclo [3.1.0] hex-2-ene, 2-methyl-5-(1-methylethyl)-	1082.88	nd	0.33	nd
13	cis-Linalool oxide	1093.07	0.45	0.37	0.45
14	Linalool	1124.25	1.06	0.47	1.06
15	Nonanal	1129.41	0.17	0.33	0.17
16	Phenylethyl Alcohol	1136.01	57.62	61.11	57.62
17	trans-Rose oxide	1153.41	nd	1.26	nd
18	9-Oxa-bicyclo [3.3.1] nona-3,6-dien-2-one	1159.34	0.28	0.42	0.28
19	Nerol oxide	1179.87	nd	0.42	nd
20	Terpinen-4-ol	1209.78	4.59	3.97	4.59
21	Bicyclo [3.3.1] nonan-2-one	1278.35	nd	0.84	nd
22	cis-Geraniol	1306.93	nd	10.13	nd
23	Pentadecane	1325.82	2.58	0.14	nd
24	Heptadecane	1345.6	nd	0.98	nd

No	Chemical name	RI	MD (%)	MK (%)	BC (%)
25	Pentadecane	1345.6	nd	nd	2.58
26	Heptadecane	1358.5	0.17	nd	0.17
27	1-Oxaspiro [4.5] dec-6-ene, 2,6,10,10-tetramethyl-	1375.14	0.39	nd	0.39
28	Hydroxylamine, O-decyl-	1383.64	0.39	nd	nd
29	Oxalic acid, isohexyl neopentyl ester	1383.64	nd	0.14	nd
30	O-Decylhydroxylamine	1383.64	nd	nd	0.39
31	2,6,10,10-Tetramethyl-1-oxaspiro[4.5]dec-6-ene	1398.44	nd	0.14	nd
32	Pentadecane	1413.78	nd	0.47	nd
33	Tridecane	1515.98	0.50	0.14	0.50
34	3-Isopropoxy-1,1,1,7,7,7-hexamethyl-3,5,5-tris (trimethylsiloxy)tetrasiloxane	1586.23	0.22	0.61	0.22
35	Eicosane	1598.17	0.95	nd	0.95
36	Nonadecane	1598.17	nd	0.33	nd

RI = Retention index; nd = not detected; Mon Dang Prasert (MD), Mon Klai Kangwon (MK); Bishop's Castle (BC)
